# Structural and functional alteration of the gut microbiota in elderly patients with hyperlipidemia

**DOI:** 10.3389/fcimb.2024.1333145

**Published:** 2024-05-15

**Authors:** Meng Xia, Yafang Xu, Huajun Li, Juan Huang, Haolin Zhou, Chuanzhou Gao, Jingyi Han

**Affiliations:** ^1^ Department of Clinical Laboratory, First Affiliated Hospital, Dalian Medical University, Dalian, China; ^2^ Department of Microecology, College of Basic Medical Sciences, Dalian Medical University, Dalian, China; ^3^ Institute of Cancer Stem Cell, Dalian Medical University, Dalian, China

**Keywords:** hyperlipidemia, gut microbiota, 16S rRNA gene high throughput sequencing, elderly people, lipid metabolism

## Abstract

**Objective:**

To investigate the structure, composition, and functions of the gut microbiota in elderly patients with hyperlipidemia.

**Methods:**

Sixteen older patients diagnosed with hyperlipidemia (M group) and 10 healthy, age-matched normal volunteers (N group) were included. These groups were further subdivided by sex into the male normal (NM, n = 5), female normal (NF, n = 5), male hyperlipidemia (MM, n = 8), and female hyperlipidemia (MF, n = 8) subgroups. Stool samples were collected for high-throughput sequencing of 16S rRNA genes. Blood samples were collected for clinical biochemical index testing.

**Results:**

Alpha- and beta-diversity analyses revealed that the structure and composition of the gut microbiota were significantly different between the M and N groups. The relative abundances of *Bacteroides*, *Parabacteroides*, *Blautia*, *Peptococcus*, and *Bifidobacterium* were significantly decreased, while those of *Lactobacillus*, *Helicobacter*, and *Desulfovibrio* were significantly higher in the M group. There were also significant sex-related differences in microbial structure between the NM and NF groups, and between the MM and MF groups. Through functional prediction with PICRUSt 2, we observed distinct between-group variations in metabolic pathways associated with the gut microbiota and their impact on the functionality of the nervous system. Pearson’s correlation coefficient was used as a distance metric to build co-abundance networks. A hypergeometric test was used to detect taxonomies with significant enrichment in specific clusters. We speculated that modules with *Muribaculaceae* and *Lachnospiraceae* as the core microbes play an important ecological role in the intestinal microbiota of the M group. The relative intestinal abundances of *Agathobacter* and *Faecalibacterium* in the M group were positively correlated with serum triglyceride and low-density lipoprotein levels, while the relative abundance of *Bifidobacterium* was negatively correlated with the serum lipoprotein a level.

## 1 Introduction

The prevalence of hyperlipidemia has increased in parallel with the development of the social economy and improvements in living standards (Teramoto et al., 2008). Hyperlipidemia is a medical condition characterized by the abnormal concentration beyond the normal range of one or more types of lipids in the bloodstream and is a result of metabolic disorder (He and Ye, 2020). Abnormal blood lipids affect normal physiological functions, and are closely related to a variety of chronic metabolic diseases (DeBose-Boyd, 2017). They are also considered a main predisposing factor of cardiovascular illnesses, including atherosclerotic disease, coronary heart disease, and myocardial infarction (Wang et al., 2013; Oliveira and Raposo, 2020).

There are about 100 trillion non-pathogenic microorganisms in the human intestine. The number of microorganisms per gram of colon contents can reach 10^12^ (Kim and Jazwinski, 2018). The gut microbial population collectively encodes millions of genes, which gives it the ability to modify and regulate the physiological functions of the host. Changes in the gut microbiota have typically been related to age, host genes, lifestyle, and epigenetic changes (Adak and Khan, 2019). There are differences in the gut microbiota of healthy people of different physiological ages. For example, the gut microbiota compositions of infants, adults, and the elderly are different (Milani et al., 2017; Salazar et al., 2017). The diversity of the gut microbiota in stool samples of children is significantly lower than that of adults (Iglesias-Vázquez et al., 2020). Studies have shown that the changes in the gut microbiota significantly with age (O’Toole and Jeffery, 2015).

Chronic metabolic diseases such as hyperlipidemia and hyperglycemia occur mostly in elderly people (Ducharme and Radhamma, 2008). Further research has revealed a close relationship between the gut microbiota and hyperlipidemia. Our previous animal experiments showed that the gut microbiota of mice with diet-induced hyperlipidemia is significantly different from that of normal mice, and that the gut microbiota also affects the metabolism of lipids and bile acids in mice (Chen et al., 2019). Another study found that material energy metabolism, the inflammatory response, and insulin resistance in the host all involve the participation of the gut microbiota (Tosti et al., 2018). Increasingly more scholars have come to believe that there is a correlation between hyperlipidemia and the gut microbiota, and the gut microbiota likely plays a role in the occurrence and development of metabolic diseases such as hyperlipidemia (Busnelli et al., 2018; Huang et al., 2019).

In this study, we used high-throughput sequencing of 16S rRNA genes in the fecal microbiota of elderly patients with hyperlipidemia and healthy elderly volunteers to examine differences in the gut microbiota between the two groups and to identify correlations between the gut microbiota and hyperlipidemia. This study may provide guidance and new ideas for the prevention and clinical treatment of hyperlipidemia.

## 2 Materials and methods

### 2.1 Participants

The M group (n = 16) comprised patients who were previously diagnosed with hyperlipidemia (total cholesterol [TC] > 5.7mmo/L) and recruited from the First Affiliated Hospital of Dalian Medical University. The normal (N) group (n = 10) comprised age-matched healthy (TC < 5.2mmol/L) volunteers. The M and N groups were subdivided by sex into the male normal (NM, n = 5), female normal (NF, n = 5), male hyperlipidemia (MM, n = 8), and female hyperlipidemia (MF group, n = 8) groups.

The inclusion criteria were as follows: 1) male or female; 2) meeting diagnostic criteria for hyperlipidemia (M group only) without receiving any lipid-lowering treatment for hyperlipidemia; and 3) body mass index (BMI) within the range of 20–30 kg/m^2^ for all participants. The exclusion criteria for all participants were as follows: 1) a history of metabolic diseases, including diabetes and thyroid disease; 2) a history of peptic diseases, including intestinal inflammatory ulcers; and 3) use of antibiotics, probiotics, prebiotics, postbiotics, or immunosuppressive agents in the previous 2 months. The study was conducted in accordance with the principles of the Declaration of Helsinki, and the study protocol was approved by the First Affiliated Hospital Ethical Committee of Dalian Medical University (approval number: PJ-KS-KY-2021–90).

### 2.2 Sample collection and DNA extraction

Blood samples were separated by centrifugation at 3000 rpm at 4°C for 20 min to obtain serum, which was used to measure levels of the following: uric acid (UA), homocysteine (HCY), fasting blood glucose (FBG), alanine aminotransferase(ALT), aspartate aminotransferase (AST), creatinine (Cre), prealbumin (PAB), albumin (ALB), globulin (GLB), total protein (TP), alkaline phosphatase (ALP), γ-glutamyl transpeptidase (γ-GT), total bilirubin (T-BIL), lipoprotein a (LPa), TC, triglycerides (TG), high -density lipoprotein (HDL), and low-density lipoprotein (LDL).

Fresh samples of feces (200 mg) were collected from each participant into a sterile container and immediately stored at −80°C until further processing. The Stool DNA Isolation Kit (Foregene, China) was used to extract genomic DNA from stool samples, in accordance with the manufacturer’s instructions.

### 2.3 16S rRNA gene amplification and sequencing

The V3-V4 hypervariable variable regions of genomic DNA samples were amplified by polymerase chain reaction (PCR) using primers 338F (5’-ACTCCTACGGGAGGCAGCA-3’) and 806R (5’-GGACTACHVGGGTWTCTAAT-3’). PCR products were subjected to 16S rRNA gene high-throughput sequencing by Shanghai Maggi Biomedical Technology Co., Ltd., using an Illumina MiSeq PE300. The 16S rRNA gene sequences were defined as one operational taxonomic unit (OTU) based on 97% similarity. The abundance-based coverage estimator (ACE) index, observed species (Sobs) index, Shannon diversity index, and Faith’s phylogenetic diversity (PD) were used to reflect the alpha diversity of samples. Principal component analysis (PCA) and principal co-ordinates analysis (PCoA) were used to analyze beta diversity. Linear discriminant analysis (LDA) was used to screen for dominant microbial communities (Huang et al., 2021). Phylogenetic Investigation of Communities by Reconstruction of Unobserved States (PICRUSt) was applied to predict functional profiles of the gut microbiota resulting from reference-based OTU picking against the Greengenes database. The predicted genes were then summarized by Kyoto Encyclopedia of Genes and Genomes (KEGG) pathway categorization. Pearson’s correlation coefficients were calculated using the difference-rich OTU, and network analysis was conducted. Gephi was used for topology analysis and visualization purposes.

### 2.4 Statistical analysis

All experimental data are presented as the mean ± standard deviation (SD). SPSS version 22 (IBM, USA) was used to analyze and process data. Statistical analysis of KEGG pathway data was performed with STAMP v2.1.3 using Welsh’s t-test (*P* < 0.05). R software version 3.5.2 was used to analyze bioinformatics results. Graph Pad Prism Version 8 (Graph Pad Software Inc., USA) was used to draw statistical charts. One-way analysis of variance (ANOVA) and Duncan’s multiple range tests were used to analyze statistical data. A *P* value < 0.05 was considered to indicate statistical significance; *P < 0.05, **P < 0.01, ***P < 0.001.

## 3 Result

### 3.1 Basic clinical information and serum biochemical indicators in elderly hyperlipidemia patients and healthy volunteers

Age and BMI were not significantly different between the M and N groups (*P* > 0.05; **Table 1**). Serum lipid levels (TC, TG, HDL, LDL, LPa) were significantly higher in the M group than in the N group (*P* < 0.05; **Table 1**). There were no significant between-group differences in other serum parameters (UA, HCY, FBG, ALT, AST, Cre, PAB, ALB, GLB, TP, ALP, γ-GT, T-BIL; *P* > 0.05; **Table 1**), indicating the comparability between the hyperlipidemia and healthy groups.

**Table 1:** Basic clinical information and biochemical indicators of the patient.

Parameters		Normal Group (n=10)	Hyperlipidemia Group (n=16)	*P*
Gender				1
Male		5	8	
Female		5	8	
Age	years	62.40 ± 7.34	66.31 ± 4.96	0.117
Body mass index (BMI)	kg/m^2^	24.52 ± 2.27	24.93 ± 2.24	0.658
Uric acid (UA)	μmol/L	311.51 ± 63.7	359.06 ± 98.43	0.188
Homocysteine (HCY)	μmol/L	10.74 ± 2.50	12.09 ± 4.16	0.367
Fasting blood glucose (FBG)	mmol/L	5.00 ± 0.51	5.15 ± 0.53	0.488
Alanine aminotransferase (ALT)	U/L	21.00 ± 7.97	19.61 ± 12.41	0.762
Aspartate aminotransferase (AST)	U/L	19.00 ± 5.75	22.54 ± 6.94	0.207
creatinine (Cre)	μmol/L	65.10 ± 9.10	66.83 ± 14.85	0.751
Prealbumin (PAB)	mg/L	247.78 ± 63.10	268.81 ± 38.41	0.371
Albumin (ALB)	g/L	41.31 ± 2.13	40.70 ± 4.90	0.717
Globulin (GLB)	g/L	23.80 ± 3.28	26.02 ± 2.95	0.103
Total Protein (TP)	g/L	65.11 ± 4.92	66.72 ± 6.75	0.532
Alkaline phosphatase (ALP)	U/L	68.00 ± 13.84	73.00 ± 17.41	0.465
γ-glutamyl transpeptidase (γ-GT)	U/L	21.00 ± 9.74	26.62 ± 19.38	0.413
Total bilirubin (T-BIL)	μmol/L	13.52 ± 5.49	11.12 ± 3.53	0.217
Total cholesterol (TC)	mmol/L	3.78 ± 0.91	6.21 ± 0.50	< 0.001
Triglyceride (TG)	mmol/L	0.99 ± 0.32	1.72 ± 0.54	0.001
High-density lipoproteins (HDL)	mmol/L	1.05 ± 0.20	1.25 ± 0.25	0.046
Low-density lipoproteins (LDL)	mmol/L	2.07 ± 0.64	3.83 ± 0.45	< 0.001
Lipoprotein a (LPa)	mg/L	127.89 ± 93.93	348.31 ± 149.79	0.001

### 3.2 Quality evaluation of DNA samples and 16S rRNA gene amplification and sequencing

Fecal DNA samples with a concentration > 50 ng/µL and a purity ratio of A260/A280 > 1.8 were used to prepare libraries and conduct sequencing (**Supplementary Table 1**). We used the extracted data volume as the abscissa and the Sobs and Shannon index values as the ordinate to draw the rarefaction curve. Curve flatness was used to determine that the amount of sequence data was sufficient. As shown in **Supplementary Figure 1**, the sparse Sobs and Shannon curves of the M and N groups both tended to be stable, indicating that the depth of the sequencing data was sufficient to cover most of the microbial information.

### 3.3 Distinctions in relative abundances of gut microbes between elderly hyperlipidemia patients and healthy volunteers

**Figure 1:**
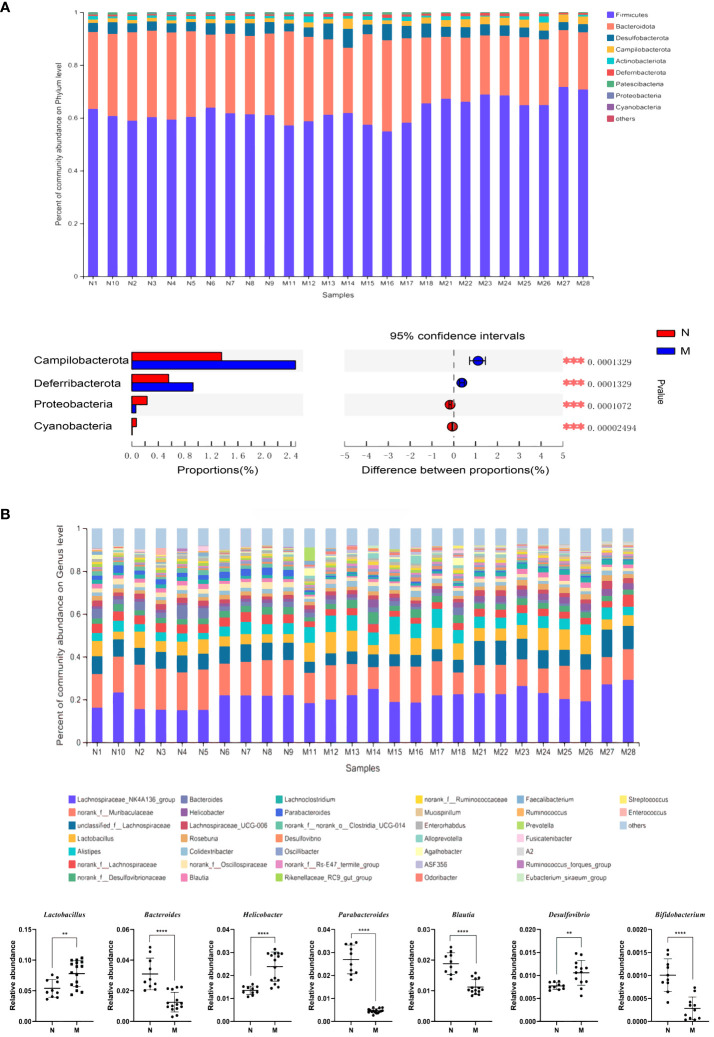
Analysis of the composition of gut microbiota in the N and M group. **(A)** Microbial distributions of different groups at the phylum level. **(B)** Microbial distributions of different groups at the genus level. N: normal group (n=10). M: hyperlipidemia group (n=16). **P<0.01, ***P<0.001, ****P<0.001 compared with the normal group.

The compositions of the gut microbiota at the phylum and genus levels are shown in **Figure 1**. There were obvious changes in the gut microbiota in the M group. At the phylum level, the top nine most prevalent phyla are shown in **Figure 1A**. Firmicutes and Bacteroidetes were the dominant phyla in all samples, followed by Desulfobacterota, Campilobacterota, Actinobacteriota and Deferribacterota. The abundances of Campilobacterota and Deferribacterota were significantly higher in the M group than in the N group (P < 0.0001). In the M group, the abundance of Proteobacteria and Cyanobacteria was significantly decreased (*P* < 0.0001). At the genus level the relative abundances of *Bacteroides* (*P* = 0.001), *Parabacteroides* (*P* < 0.0001), *Blautia* (*P* < 0.0001), *Peptococcus* (*P* < 0.0001), and *Bifidobacterium* (*P <* 0.0001) were significantly decreased, whereas those of *Lactobacillus* (*P* = 0.004), *Helicobacter*, and *Desulfovibrio* (*P* = 0.004) were significantly increased in the M group compared with those in the N group (**Figure 1B**).

### 3.4 Distinctly different patterns of the gut microbial interactions between in elderly hyperlipidemia patients with hyperlipidemia and normal healthy volunteers

**Figure 2:**
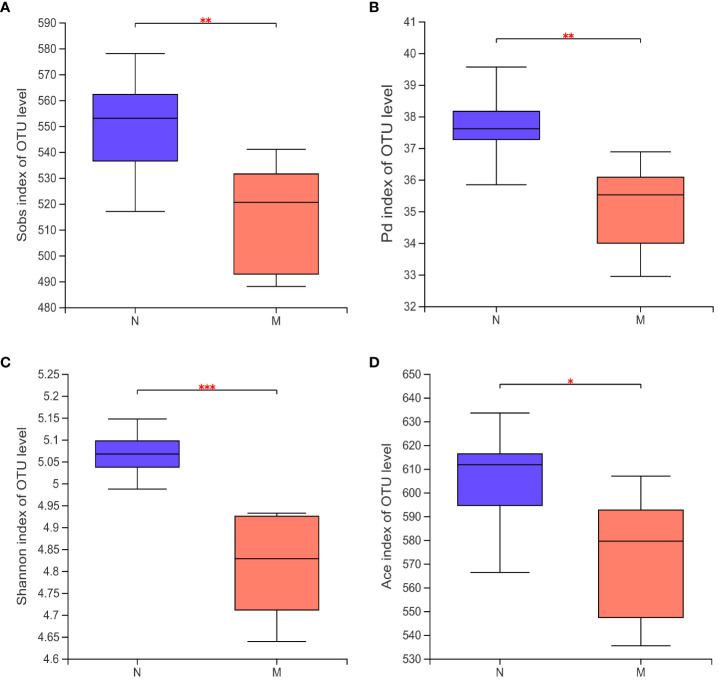
Alpha diversity of the gut microbiota in the N and M group. **(A)** Sobs index of OUT level. **(B)** Pd index of OUT level. **(C)** Shannon index of OUT level. **(D)** Ace index of OUT level. N: normal group (n=10). M: hyperlipidemia group (n=16). *P<0.05, **P<0.01, ***P<0.001, compared with the normal group.

Alpha-diversity analyses of the gut microbiota in the N and M groups are shown in **Figures 2A–D**. The Sobs (*P* = 0.008) and Shannon (*P* = 0.047) indices based on Kruskal–Wallis analysis showed that the richness and diversity of the gut microbiota were significantly lower in the M group compared with those in the N group. The ACE index (*P* = 0.023) and Faith’s PD (*P* = 0.002) metrics further confirmed this result.

**Figure 3:**
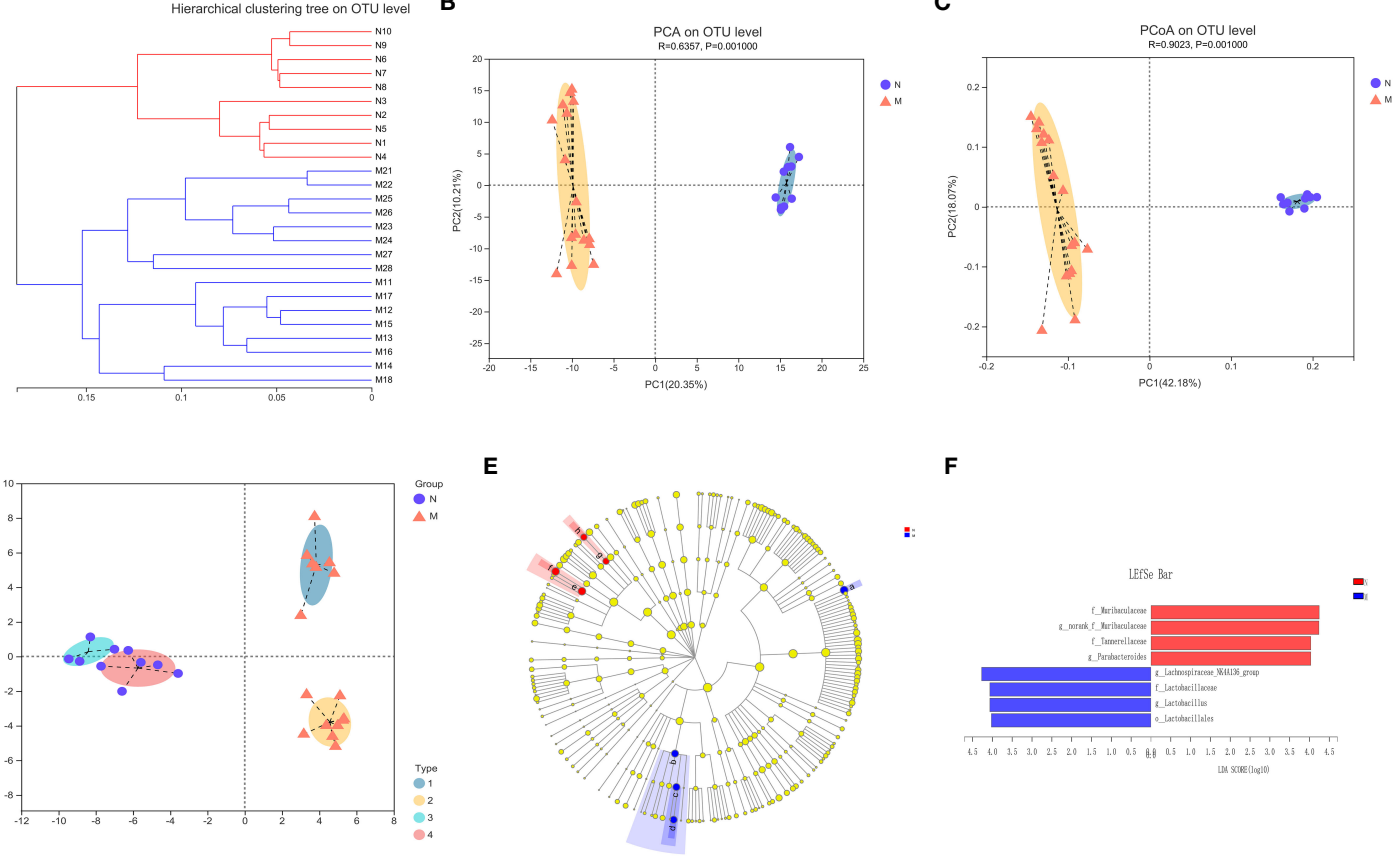
Analysis of the structure and communities of gut microbiota in the N and M group. **(A)** The microbial composition with the cluster at the OTU level. **(B)** Principal component analysis (PCA) with the cluster. **(C)** Principal co-ordinates analysis (PCoA) with cluster. **(D)** Bacteria typing analysis. **(E)** Cladogram. **(F)** LDA distribution. N: normal group (n=10). M: hyperlipidemia group (n=16).

**Figure 3A** illustrates the beta-diversity of the gut microbiota in the two groups as hierarchical cluster trees at the OTU level based on Bray–Curtis dissimilarity (**Figure 3A**). PCA and PCoA revealed that the microbial structures of the M and N groups were significantly different (**Figures 3B, C**), supporting the difference in gut microbiota composition between healthy individuals and hyperlipidemia patients shown above. Next, we used LDA of effect size (LEfSe) to further screen for dominant microbial communities between the two groups at the genus level. The results indicated that *Lactobacillus* was enriched in the M group, whereas *Parabacteroides* and *Lachnospiraceae-NK4A136* were enriched in the N group (**Figures 3E, F**). Bacterial typing analysis categorized the gut microbiota of the N and M groups into four types (**Figure 3D**). Coincidentally, when sex was considered as a grouping element, elderly healthy participants and hyperlipidemia patients were divided into four groups.

### 3.5 Influence of sex differences on gut microbiota characteristics between in elderly hyperlipidemia patients with and hyperlipidemia and normal healthy volunteers

**Figure 4:**
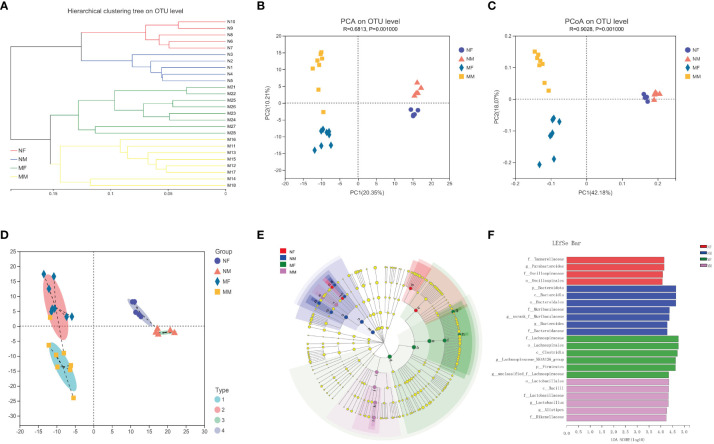
Analysis of the structure and communities of gut microbiota in the NF, NM, MF and MM groups. **(A)** microbial composition with the cluster at the OTU level. **(B)** principal component analysis (PCA) with cluster. **(C)** principal co-ordinates analysis (PCoA) with cluster. **(D)** bacteria typing analysis. **(E)** cladogram. **(F)** LDA distribution. NF group, the female normal group; NM group, the male normal group; MM group, the hyperlipidemia male patient group; MF group, the hyperlipidemia female patient group. NM, male normal group (n=5); NF, female normal group (n=5); MM, the hyperlipidemia male patient group (n=8); MF, hyperlipidemia female patient group (n=8).

To further investigate the potential impact of sex on the distribution of intestinal microbes, we analyzed the male and female subgroups of elderly healthy volunteers and hyperlipidemia patients (**Figure 4**). There were significant differences in gut microbial diversity between the male and female subgroups within both the N and M groups, with significant clustering observed on PCA and PCoA. Among the elderly healthy volunteers, the NF subgroup was rich in *Parabacteroides*, whereas the NM subgroup was rich in *Bacteroides*. Among the elderly patients with hyperlipidemia, *Lachnospiraceae-NK4A136* was enriched in the MF subgroup, whereas *Lactobacillus* and *Alistipes* were enriched in the MM subgroup.

### 3.6 Potential functions and identification of co-abundance networks of OTUs in the two groups

**Figure 5:**
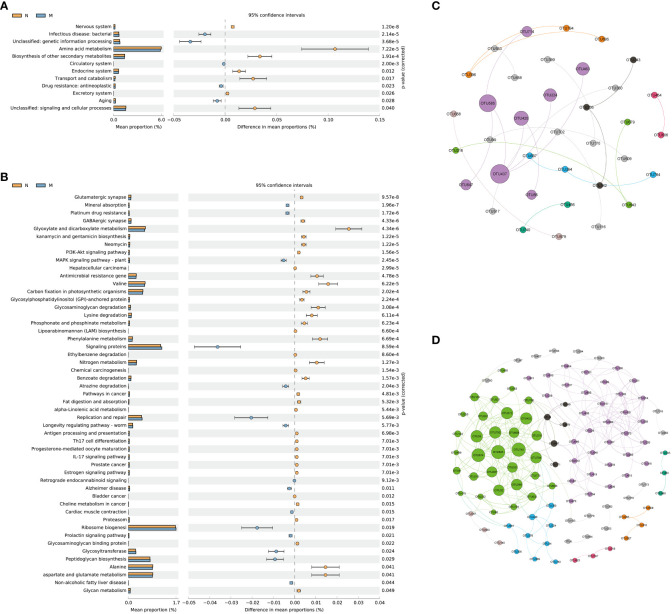
Functional prediction and co-abundance networks. **(A)** STAMP analysis for the inferred metabolic pathway in level 2. **(B)** STAMP analysis for the inferred metabolic pathway in level 3. **(C)** The co-abundance networks of the N group. **(D)** The co-abundance networks of the M group. N: normal group (n=10). M: hyperlipidemia group (n=16).

KEGG level 2 functional pathway analysis indicated that the nervous system, amino acid metabolism, biosynthesis of other secondary metabolites, endocrine system, transport and catabolism, excretory system, signaling, and cellular processes were significantly reduced in the M group compared to those in the N group. Conversely, infectious disease (bacterial), genetic information processing, circulatory system, drug resistance (antineoplastic), and aging pathways were significantly increased in the M group compared to those in the N group (**Figure 5A**).

At level 3, a total of 51 pathways exhibited significant differences between the two groups. Among the top 10 dominant KEGG pathways, the relative abundances of the glutamatergic synapse, GABAergic synapse, kanamycin and gentamicin biosynthesis, neomycin, the PI3K-Akt signaling pathway, and hepatocellular carcinoma were significantly lower in the M group than in the N group. Conversely, mineral absorption, platinum drug resistance, and the MAPK signaling pathway (plant) displayed significantly higher abundances in the M group than in the N group (**Figure 5B**).

Functional prediction with PICRUSt 2 revealed distinct variations in metabolic pathways associated with the gut microbiota and their impacts on the functionality of the nervous system between the two groups. Sequence-based characterization, which focuses on how individual taxa within the gut microbiome relate to the host, does not reveal the complex interactions that take place between taxa within microbial communities. Microbes cooperate in networks to provide critical nutrients for each other’s growth and survival. The identification of microbial communities is important for understanding their biological impact on the human body, but is hampered by our inability to culture most microbes. We hypothesized that generating OTU abundance modules from lists of differential OTUs between the two groups would allow us to identify associations with differential changes in function in elderly hyperlipidemia patients.

**Figures 5C** and **D** illustrate the more intricate network structure in group M compared to that in the N group, implying reduced stability of the intestinal microbiota in elderly patients with hyperlipidemia. Subsequent examination of the modules revealed that those containing *Muribaculaceae* and *Lachnospiraceae* as central microbial species hold significant ecological relevance in elderly patients with hyperlipidemia.

### 3.7 Correlation between altered gut microbiota and blood lipids in elderly patients with hyperlipidemia

**Figure 6:**
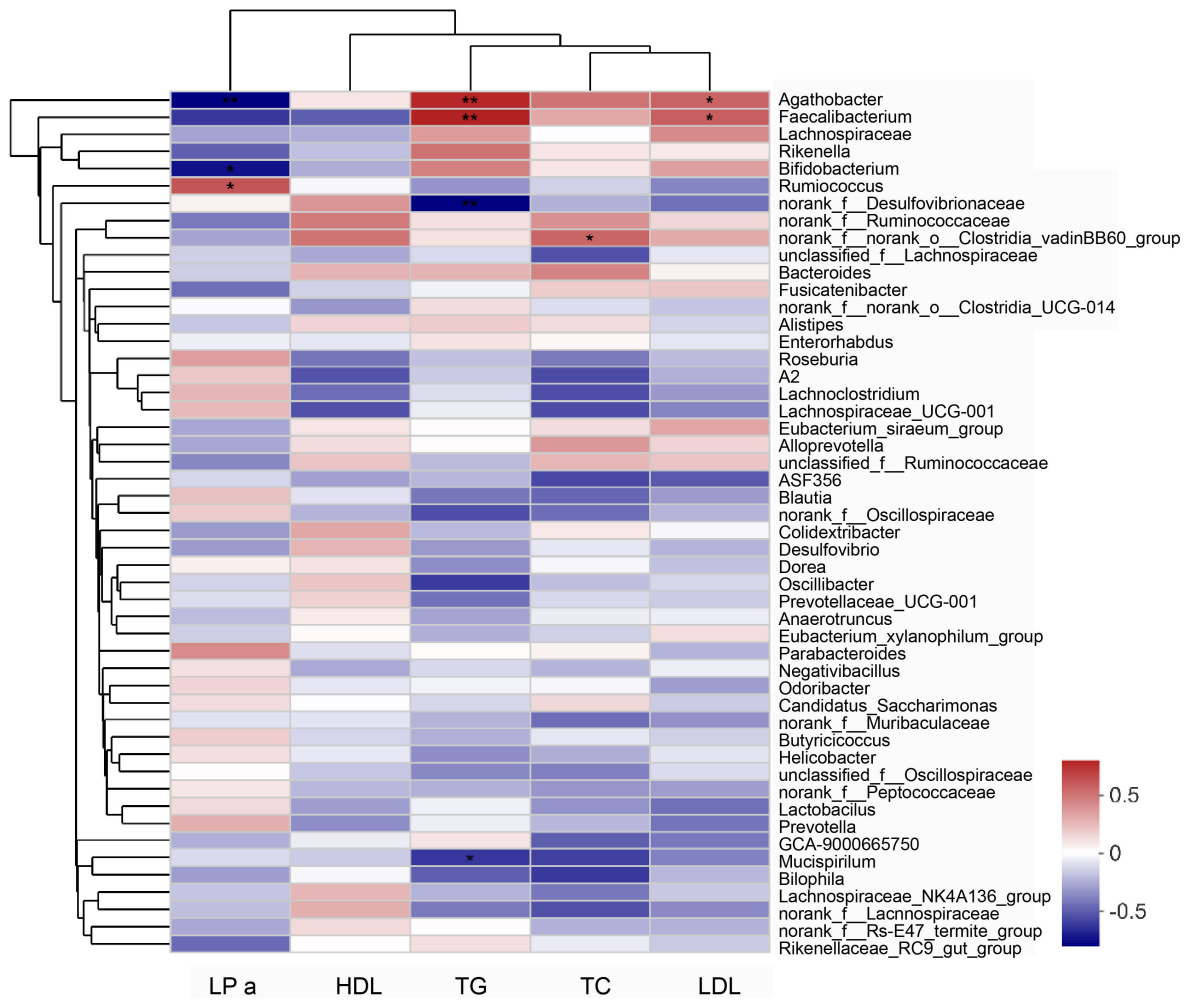
Analysis of the correlation between serum lipid parameters and gut microbiota in patients with hyperlipidemia. “0” means no correlation; “0-(0.5)” means positive correlation; “0-(-0.5)” means negative correlation. Data were analyzed by Spearman test. *P<0.05, **P<0.01. LPa, Lipoprotein a; TC, total cholesterol; TG, triglycerides; HDL, High-density lipoprotein; LDL, Low-density lipoprotein.

The Spearman’s correlation coefficient was used to assess the relationship between the gut microbiota and blood lipid levels. In the correlation heat map shown in **Figure 6**, blue represents positive correlations between bacteria and serum parameters, while red represents negative correlations (**Figure 6**). Specifically, *Agathobacter* and *Faecalibacterium* in the intestine of elderly patients with hyperlipidemia were positively correlated with serum TG and LDL levels, while *Bifidobacterium* was negatively correlated with the serum LPa level (**Table 2**).

**Table 2:** Correlation between serum lipid parameters and gut microbiota in patients with hyperlipidemia.

Parameters	*Desulfovibrionaceae*		*Mucispirillum*		*Agathobacter*		*Faecalibacterium*		*Ruminococcus*		*Bifidobacterium*	
	Correlation	*P*-value	Correlation	*P*-value	Correlation	*P*-value	Correlation	*P*-value	Correlation	*P*-value	Correlation	*P*-value
TC	-0.174	0.517	-0.474	0.064	0.461	0.073	0.302	0.256	-0.090	0.741	0.115	0.671
TG	-0.643	0.007	-0.500	0.048	0.696	0.003	0.711	0.002	-0.252	0.347	0.423	0.102
HDL	0.356	0.175	-0.104	0.700	0.113	0.676	-0.393	0.132	0.009	0.974	-0.188	0.486
LDL	-0.343	0.193	-0.299	0.261	0.511	0.043	0.522	0.038	-0.291	0.274	0.329	0.214
LPa	0.076	0.778	-0.068	0.803	-0.641	0.007	-0.494	0.052	0.556	0.025	-0.595	0.015

Correlation coefficient greater than 0, positive correlation between bacteria and serum factor; less than 0, negative correlation between bacteria and serum factor. Data (n=16) were analyzed by the Spearman test.

## 4 Discussion

In recent years, increasingly more studies have shown that the normal gut microbiota is inextricably linked with the metabolism of the body, and may regulate blood lipids (Patterson et al., 2016; Hills et al., 2019; Silva et al., 2020). In this research, we focused on exploring hyperlipidemia-related changes in the gut microbiota of elderly men and women. We found that the relative abundances of *Bacteroides, Parabacteroides*, *Blautia*, *Peptococcus*, and *Bifidobacterium* in the gut microbiota were significantly reduced, while those of *Lactobacillus, Helicobacter*, and *Desulfovibrio* were significantly increased with hyperlipidemia. Similar results have been observed in previous studies of patients with dyslipidemia, reporting varying degrees of changes in gut microbes, including *Lactobacillus*, *Bifidobacterium*, *Bacteroidetes*, *Enterococcus*, *Enterobacteriaceae* species, *Clostridium*, in which the proportion of *Lactobacillus* was upregulated (Song et al., 2017; Gargari et al., 2018; Jia et al., 2021; Guo et al., 2022).

Under normal circumstances, certain common intestinal bacteria produce cholesterol oxidase to accelerate the degradation of cholesterol, thereby participating in maintaining the normal level of cholesterol in the body. In addition, beneficial bacteria in the intestines, such as *Clostridium*, *Bifidobacterium*, *Bacteroides*, and *Enterococcus* produce bound bile acid hydrolase, which converts bound bile acid into free bile acid (Cai et al., 2022; Pushpass et al., 2022). However, under conditions of hyperlipidemia, the living environment of the gut microbiota undergoes significant changes, with the low relative abundances of *Bifidobacterium* and *Bacteroides* leading to an increase in accumulated cholesterol.

Some studies reporting the effects of lactic acid bacteria and fermented dairy products on blood lipids found that lactic acid bacteria, including *Bifidobacteria* lower serum cholesterol levels (Schoeler and Caesar, 2019). Most of these bacteria act on TG, TC, HDL, and LDL in the serum, thereby reducing blood lipids. It has also been reported that the content of TG in the serum of patients with hyperlipidemia is significantly negatively correlated with *Bifidobacterium* and *Lactobacillus*, and positively correlated with *Enterobacteriaceae* and *Enterococcus* (Osman et al., 2005). Our study found that the abundances of *Agathobacter* and *Faecalibacterium* in the intestines of elderly patients with hyperlipidemia were positively correlated with serum TG and LDL levels, while *Bifidobacterium* abundance was negatively correlated with the serum LPa level.

Gut microbial diversity changes throughout the human life span and is known to be associated with the sex of the host. The latest research has shown that many characteristics, including sex, age, TG and uric acid levels, obesity, and lifestyle, have significant impacts on the gut microbiota (Portune et al., 2017; Just et al., 2018; Ma et al., 2020). One study found that the gut microbiota is obviously dependent on sex, with higher alpha-diversity in women than in men (de la Cuesta-Zuluaga et al., 2019). Consistently, our research showed significant sex-related impacts on the microbiota structure and diversity of patients with hyperlipidemia. Beta-diversity analyses performed on the Bray-Curtis distance matrix showed different hierarchical cluster trees at the OTU level between healthy elderly males and females and between elderly males and females with hyperlipidemia. PCA and PCoA also showed significant differences in the microbial structure between elderly male and female patients with hyperlipidemia.

Numerous studies have established correlations between abundances of *Muribaculaceae* and *Lachnospiraceae* and metabolic diseases. *Muribaculaceae* bacteria are involved in the synthesis of short-chain fatty acids and influence the host’s metabolic function (Song et al., 2022; Bai et al., 2023). *Lachnospiraceae* bacteria possess the capacity to reduce inflammation and have been associated with cancer and neurological diseases (Shen et al., 2021; Du et al., 2023).

This study has one main limitation. While our aim was to explore sex- and hyperlipidemia-related changes in the gut microbiota in elderly patients, the number of participants was limited. We are currently conducting animal and cell experiments to further validate and explore the underlying mechanisms of our results.

In summary, the structure and composition of the gut microbiota in elderly patients with hyperlipidemia appear to undergo significant changes that are closely related to serum lipid levels and metabolic pathway activity. We also discovered sex-related differences in the distribution of the gut microbiota. Interestingly, modules with *Muribaculaceae* and *Lachnospiraceae* as the core microbes played an important ecological role in the gut microbiota of elderly patients with hyperlipidemia. Consideration of the relationship between the gut microbiota and hyperlipidemia should include the impact of sex differences.

## Data availability statement

The datasets presented in this study can be found in online repositories. The names of the repository/repositories and accession number(s) can be found below: NCBI BioProject ID is PRJNA1063629.

## Ethics statement

The studies involving humans were conducted in accordance with the principles of the Declaration of Helsinki and approved by the ethics committee of the First Affiliated Hospital Ethical Committee of Dalian Medical University. Approval Number: PJ-KS-KY-2021-90. The studies were conducted in accordance with the local legislation and institutional requirements. The participants provided their written informed consent to participate in this study. Written informed consent was obtained from the individual(s) for the publication of any potentially identifiable images or data included in this article. The studies were conducted in accordance with the local legislation and institutional requirements. Written informed consent for participation in this study was provided by the participants’ legal guardians/next of kin. Written informed consent was obtained from the individual(s) for the publication of any potentially identifiable images or data included in this article.

## Author contributions

MX: Writing – original draft, Writing – review & editing. YX: Writing – review & editing. HL: Writing – review & editing, Methodology, Conceptualization. JuH: Writing – review & editing, Data curation, Writing – original draft. HZ: Data curation, Writing – review & editing, Writing – original draft. CG: Writing – review & editing. JiH: Writing – original draft, Writing – review & editing, Conceptualization.
