# Supplementary material for: Structural and functional alteration of the gut microbiota in elderly patients with hyperlipidemia
Source: Front Cell Infect Microbiol. 2024 May 15;14:1333145. doi: 10.3389/fcimb.2024.1333145 (PMC11133514; doi:10.3389/fcimb.2024.1333145)
Supplement: Supplementary file 1 [file DataSheet_1.docx]

**Supplementary Table 1** The quality of DNA amplification and sequencing.

| Sample | Concentration (ng/µl) | OD260/280 | Reads | OTUs |
| --- | --- | --- | --- | --- |
| N1 | 66.30 | 2.08 | 35668 | 570 |
| N2 | 66.80 | 2.03 | 40853 | 546 |
| N3 | 65.40 | 2.09 | 35548 | 600 |
| N4 | 67.80 | 2.10 | 37053 | 574 |
| N5 | 65.00 | 2.07 | 37660 | 550 |
| N6 | 82.00 | 2.06 | 35394 | 592 |
| N7 | 71.70 | 2.06 | 37082 | 570 |
| N8 | 74.00 | 2.05 | 40196 | 552 |
| N9 | 75.00 | 2.08 | 32326 | 570 |
| N10 | 78.90 | 2.08 | 30432 | 553 |
| M11 | 123.90 | 2.09 | 34905 | 555 |
| M12 | 113.90 | 2.09 | 34263 | 563 |
| M13 | 114.30 | 2.08 | 33769 | 528 |
| M14 | 142.50 | 2.12 | 34854 | 507 |
| M15 | 114.50 | 2.10 | 31619 | 591 |
| M16 | 115.40 | 2.09 | 31962 | 562 |
| M17 | 120.00 | 2.09 | 34510 | 566 |
| M18 | 120.30 | 2.09 | 31567 | 550 |
| M21 | 118.00 | 2.05 | 37623 | 555 |
| M22 | 117.40 | 2.06 | 36453 | 563 |
| M23 | 123.40 | 2.07 | 32637 | 499 |
| M24 | 117.10 | 2.08 | 35491 | 581 |
| M25 | 94.30 | 2.07 | 35189 | 550 |
| M26 | 96.80 | 2.07 | 33574 | 583 |
| M27 | 99.80 | 2.06 | 28616 | 488 |
| M28 | 96.10 | 2.04 | 37286 | 545 |

N: the normal group (n=10); M: the hyperlipidemia group (n=16).


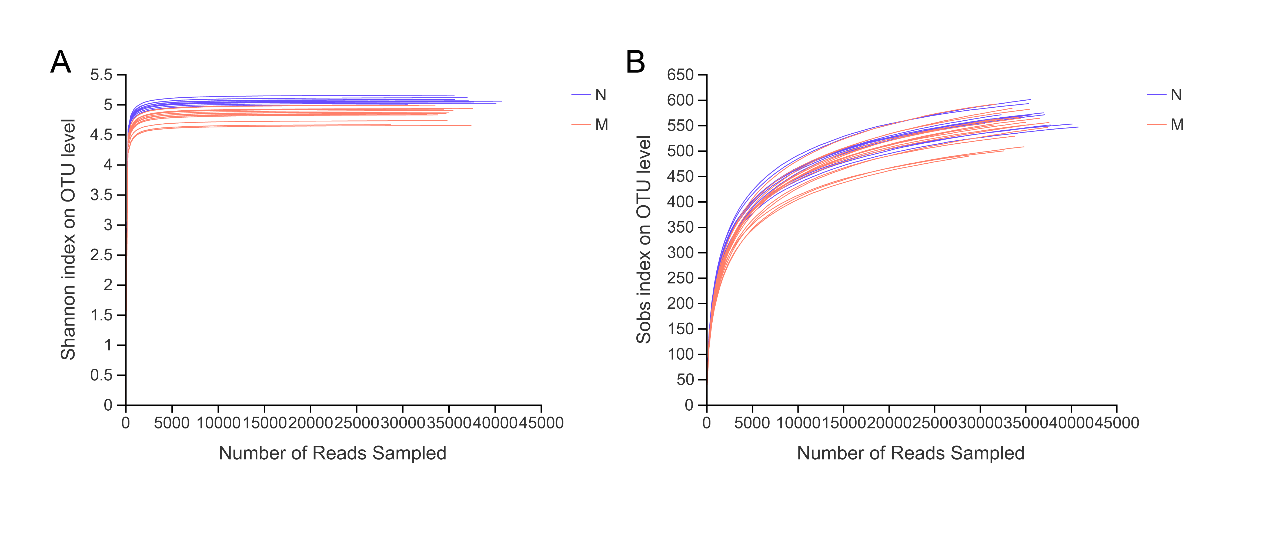
 **Supplementary Figure 1** The rarefaction curve of random sequences per sample and their corresponding number of Shannon index (A) and Sobs (B). N: the normal group (n=10); M: the hyperlipidemia group (n=16).
